# *Persea Americana* Agro-Industrial Waste Biorefinery for Sustainable High-Value-Added Products

**DOI:** 10.3390/polym13111727

**Published:** 2021-05-25

**Authors:** Anthony Mora-Sandí, Abigail Ramírez-González, Luis Castillo-Henríquez, Mary Lopretti-Correa, José Roberto Vega-Baudrit

**Affiliations:** 1 School of Chemistry, National University of Costa Rica (UNA), Heredia 86-3000, Costa Rica; tonyms1596@gmail.com (A.M.-S.); gonzalez.ramirez.abigail16@gmail.com (A.R.-G.); 2 National Laboratory of Nanotechnology (LANOTEC), National Center for High Technology (CeNAT), San José 1174-1200, Costa Rica; luis.castillohenriquez@ucr.ac.cr; 3 Faculty of Pharmacy, University of Costa Rica, San José 11501-2060, Costa Rica; 4 Nuclear Research Center, Faculty of Science, Universidad de la República (UdelaR), Montevideo 11300, Uruguay; mary@cin.edu.uy

**Keywords:** avocado, biofuel, biomass, biopolymers, by-product, renewable energy, waste valorization

## Abstract

Significant problems have arisen in recent years, such as global warming and hunger. These complications are related to the depletion and exploitation of natural resources, as well as environmental pollution. In this context, bioprocesses and biorefinery can be used to manage agro-industrial wastes for obtaining high-value-added products. A large number of by-products are composed of lignin and cellulose, having the potential to be exploited sustainably for chemical and biological conversion. The biorefinery of agro-industrial wastes has applications in many fields, such as pharmaceuticals, medicine, material engineering, and environmental remediation. A comprehensive approach has been developed toward the agro-industrial management of avocado (*Persea americana*) biomass waste, which can be transformed into high-value-added products to mitigate global warming, save non-renewable energy, and contribute to health and science. Therefore, this work presents a comprehensive review on avocado fruit waste biorefinery and its possible applications as biofuel, as drugs, as bioplastics, in the environmental field, and in emerging nanotechnological opportunities for economic and scientific growth.

## 1. Introduction

For years, the world has focused on economic development without any concern or planning regarding the exploitation of natural resources [1]. Currently, significant problems have arisen due to that behavior, such as global warming, climate change, and hunger. In addition, overpopulation generates instability in economic models, forcing societies to find sustainable ways for development, such as biobased industries that can mitigate pollution and the depletion of natural sources [2].

One strategy that impacts all fields is the recovery of agro-industrial wastes. This assessment includes the implementation of biorefineries, which complement or replace current oil refineries, thus reducing our impact on the environment [3]. Biorefinery involves converting biomass through various technologies to obtain an extensive list of high-value-added products and energy from a biobased raw material [4]. Many biotechnological bioprocesses have been developed to optimize energy consumption and reduce the amount of waste [2,5]. However, it has been difficult for innovative and sustainable industries to move towards the concepts of green chemistry, cleaner production, and zero waste [6].

Generally, agro-industrial waste is mainly composed of lignocellulosic biomass (LCB), which has gained attention in material and chemical engineering. Due to its outstanding properties (e.g., mechanical, thermal, renewability, wide availability, non-toxicity, low cost, and biodegradability) it is regarded as a novel alternative for obtaining high-value-added products [7]. Charpentier et al. recently used empty palm fruit bunch fiber (EPFBF), a lignocellulosic biomass by-product of palm oil production, for obtaining fermentable sugars. In addition, pretreated EPFBF hydrolysate was subjected to simultaneous saccharification and fermentation for bioethanol production [8].

Moreover, avocado (*Persea americana*) industrial wastes are being addressed with great importance [9,10]. This native tree of Mesoamerica has the largest production areas concentrated in Central and South America, while its consumption is increasing worldwide [11,12]. According to the average production between 1994 and 2019, the top 10 avocado producers were Mexico, the Dominican Republic, Indonesia, Colombia, Peru, United States, Brazil, Chile, Kenya, and Malawi. In 2019, the Food and Agriculture Organization of the United Nations (FAO) reported a world production of 7.0 million tons, of which approximately half was lost throughout the production chain [12].

The biorefinery of this fruit waste is mandatory to provide a solution to this problem and to the need for creating sustainable economic growth. In this sense, Trujillo et al. compared three different avocado fruit sizes (small, medium, and large) regarding their total phenolic (TPC) and flavonoid contents (TFC). The study reported higher TPC, TFC, and antioxidant and enzymatic activities in avocado peel extract from the small size group, while these parameters were significantly lower in the other groups. These results suggest avocado by-products’ suitability for biorefinery applications [13].

Furthermore, the valorization of avocado fruit by-products within a biorefinery frame allows for obtaining different bioactive compounds for therapeutic applications [14]. Additionally, as stated by Bayón et al., biopolymers are abundant in agro-industrial wastes. This variety of molecules presented in avocado residues can be used for drug delivery applications, tissue engineering, and other health-associated technologies [15]. Therefore, this review presents a comprehensive approach to the biorefinery of the agro-industrial wastes from *Persea americana* to obtain products with high-value-added, highlighting their applications as biofuels, bioenergy, bioactive compounds, and biopolymers with nanotechnological functions.

## 2. Applications of Avocado Agro-Industrial Wastes

### 2.1. Waste and Pretreatments

**Figure 1:**
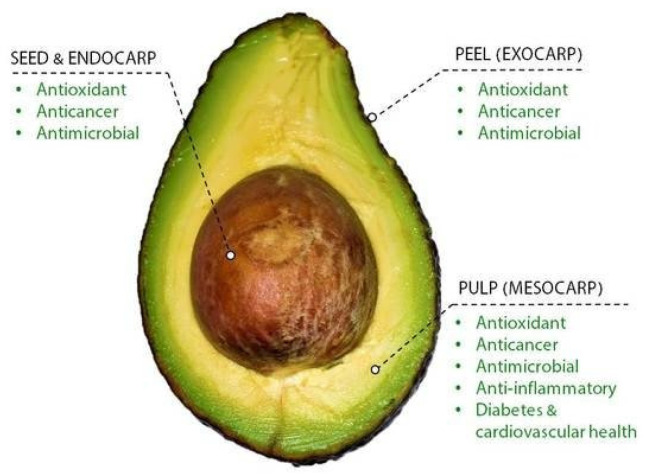
Avocado parts and properties. Reprinted with permission from Bhuyan et al. The Odyssey of Bioactive Compounds in Avocado (Persea americana) and Their Health Benefits. *Antioxidants*. Copyright (2019) MDPI [21].

An average avocado fruit weighs approximately 150–400 g [16,17]. This fruit is made up of exocarp (peel), mesocarp (pulp), endocarp, and seed (Figure 1). Mesocarp is the most abundant, representing between 52.9 and 81.3% of the fruit mass, which is in great demand because of its properties and high nutritional value [18,19]. Avocado provides vitamins, such as A, B, D, and E, that can be used for different applications (e.g., pharmaceutical, nutritional, and cosmetic industries) [19,20,21].

On the other hand, the peel and seed are not often used in the food industry, thus becoming an excessive amount of waste. These by-products usually represent between 21–30% of the avocado mass and are full of biopolymers and raw materials that constitute a great source of lignin and cellulose [22,23]. Lignin is an aromatic polymer and the second most abundant on earth after cellulose. It is usually burned to generate heat, but in biorefineries, the conversion of LCB into liquid transportation fuels generates more lignin than needed to power the process [24]. In addition, lignin makes the biopolymeric structure highly resistant to solubilization, which inhibits cellulose hydrolysis and thus causes an issue for its isolation [25,26,27]. Therefore, special efforts are underway to transform this excess into high-value-added products [24].

**Figure 2:**
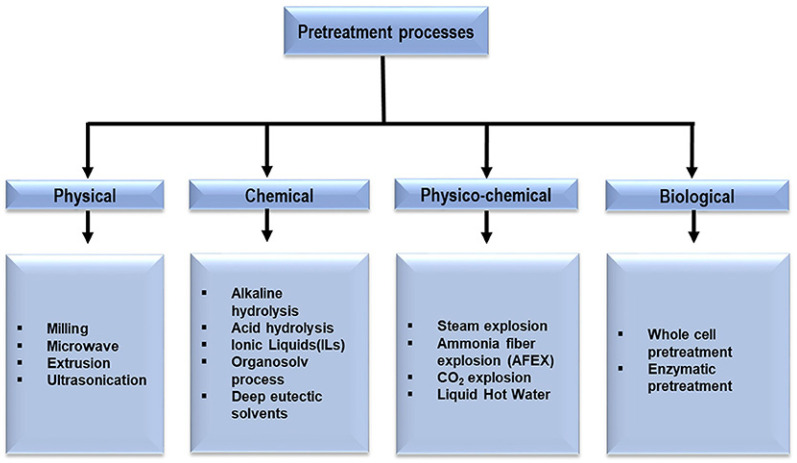
Flow chart diagram of pretreatment processes. Reprinted with permission from Baruah et al. Recent Trends in the Pretreatment of Lignocellulosic Biomass for Value-Added Products. *Frontiers in Energy Research*. Copyright (2018) Frontiers [28].

Given the above, avocado fruit wastes and LCB demand physical, chemical, and biological pretreatments (Figure 2) that ease handling and improve process efficiency significantly [28,29]. It is relevant to consider that LCB’s crystalline structure, its lignification degree, and the complexity of its cellwall constituents create a resistance to chemical and biological breakdown, known as biomass recalcitrance [30,31]. As a consequence, it is imperative to execute a pretreatment technique that allows the disruption of LCB recalcitrant structure [32,33].

In the first place, physical pretreatment is necessary before any other pretreatment or operation [28]. This action aims to reduce the particle size, which produces an increase in surface area, incrementing the accessible surface, which in turn, improves hydrolysis due to better heat and mass transfer and, consequently, the process of obtaining high-value-added products [34,35]. Although these methods are considered eco-friendly, reducing the polymerization degree, as well as the crystallinity, imply high energy consumption [36].

The most common physical pretreatments include milling, extrusion, microwaves, and ultrasonication [28]. Milling is mostly employed for reducing crystallinity and, in particular, the particle size, since it has been demonstrated that a biomass below 0.4 mm does not interfere with hydrolysis [37]. Extrusion is commonly employed for pretreating LCBs. In these methods, raw materials are passed through a tight barrel that is heated above 300 °C. The high temperature combined with the shear forces produced by the screws that spin into the barrel disrupt the LCB recalcitrant structure [38,39,40].

Regarding microwave pretreatment, the biomass sample is immersed in dilute alkalis, and it is then subjected to microwaves exposure for 5–20 min. The method is energy-efficient, easy to perform, and possesses a high-heating capacity in a short time [41,42]. Duan et al. evaluated the effects of microwave temperature, microwave time, and hydrochloric acid concentration on different physicochemical properties from pure lignin. The study reported better properties for the pretreated samples in contrast to raw lignin, allowing for the production of bio-oil of excellent quality [43].

Ultrasonication generates the disintegration of biomass as a result of cavitation, which produces the significant shear forces exerted by the liquid phase. This process allows isolating cellulose, hemicellulose, and lignin [44,45,46]. Other possible physical pretreatments implement radiation (e.g., gamma rays, electron beams), as well as thermal processes (e.g., pyrolysis, roasting, or steam explosions) [47,48].

On the other hand, chemical pretreatments focus on fractioning biomass through hydrolysis mediated by ionic or organic solvents and alkaline or acidic solution exposure [28]. Ionic liquids (IL) are green solvents composed of cations and anions and present a melting point below 100 °C. During the pretreatment, the charged species promote lignin and cellulose solubilization by interrupting intra-and intermolecular hydrogen bonding in these compounds [49,50]. Nevertheless, further research is required for developing IL with no or low toxicity to enzymes as well as low-cost recovery technologies for these solvents [51].

The organosolv pretreatment uses organic solvents or their mixtures with water to break down the internal bonds between lignin and hemicellulose, which allows obtaining a pure cellulose residue. The delignification causes an increase in pore volume and the surface area of cellulose, enhancing the accessibility for enzymatic hydrolysis and saccharification [52,53]. Among the most widely used organic solvents are ethanol, methanol, acetone, and ethylene glycol [54].

Alkali pretreatment attempts to solubilize lignin in alkali solution, usually made of hydroxides (e.g., sodium, potassium, calcium, and ammonium) [55]. In these processes, several events take place. First, saponification causes cleavage of intermolecular ester linkages between lignin and hemicellulose, resulting in their solubilization [56]. In addition, cellulose swelling reduces crystallinity and the degree of polymerization, which increases the surface area available to enzymatic hydrolysis [57,58]. The combination of this pretreatment with organic solvents or aqueous solutions enhances the process. Zhong et al. employed a diluted sodium hydroxide (NaOH)-aided methanol organosolv pretreatment for the efficient exploitation of LCB from xylose residues. This approach resulted in a more efficient delignification of 86.7% and the recovery of highly pure lignin [59].

On the other hand, acid pretreatment is based on the vulnerability of glucosidic bonds between hemicellulose and cellulose to be broken by acids, causing the breakdown of long chains into sugar monomers [60,61]. This approach usually employs sulfuric, hydrochloric, nitric, phosphoric, and acetic acids as substrates. Although it is very useful for enhancing cellulose availability and biodegradability, it is not effective in dissolving lignin, only for disrupting it [62,63].

Physicochemical pretreatment methods employ both chemical and physical techniques for disrupting LCB structure [64]. These methods can make use of hydrothermal pretreatment (steam explosion and liquid hot water) to promote the separation of the lignocellulosic matrix [65,66,67,68]. In addition, ammonia fiber explosion subjects biomass material to liquid anhydrous ammonia under high pressures, followed by a rapid depressurization of the system. The degree of disruption will depend on the temperature applied, which is usually between 60–100 °C and affects the rapidness of the ammonia vaporization [69].

Finally, biological pretreatments are based on exoenzyme-mediated decomposition or hydrolysis and saccharification. It must be a solid or liquid substrate fermentation by whole-cell systems or solely enzymatic digestion. The technique presents different advantages since it is eco-friendly, consumes less energy, and there is no inhibitor formation during the process [70,71].

Furthermore, the residues obtained by these pretreatment techniques contain essential oils, bioactive compounds, fibers, and carbon sources, giving them the potential to be exploited sustainably for chemical and biological conversion for diverse industrial fields [72].

### 2.2. Potential Biorefinery

The annual amount of biomass waste generated by agro-industrial activity is estimated at 50 billion tons [16]. This biomass can be converted into different raw materials that will be further processed in biorefineries. The previous fact demands changes in current economic industrial strategies, as well as the development of suitable and sustainable biorefinery models [73,74].

Bioactive and high-value-added products have been obtained by biorefinery of different tropical fruits [75]. Melo et al. employed an organic solvent extraction for isolating phenolic compounds such as anthocyanins and flavonoids from açaí (*Euterpe oleracea*) seeds [76]. Rezende et al. also obtained anthocyanins from Acerola (*Malpighia emarginata*) seed and peel through an ultrasound-assisted extraction with ethanol as a solvent [77]. Banana (*Musa paradisiaca*) peel biorefinery was studied by Fu et al. who were able to recover phenolic compounds through successive extractions with organic solvents [78]. In addition, Lima et al. and Mugwagma et al. used guava (*Psidium guajava*) seeds and mango (*Mangifera indica*) peel, respectively, for obtaining phenolic compounds through a hydroalcoholic extraction [79,80].

In this sense, the valorization of avocado wastes is also of great interest, not only because of the high amount of by-products generated by the activity but also due to the opportunities for obtaining bioenergy, biofuels, and other marketable products [81,82]. Dávila et al. stated in their work the presence (%wt) of cellulose (26–38%), hemicellulose (24–26%), and lignin (4%) in the avocado seed. The authors also reported the same components in peel but with certain differences in the content, where cellulose is greatly reduced (6–7%), hemicellulose is doubled (46–50%), and lignin is halved (2%) in comparison to seed [83]. As mentioned before, the rich content of LCB fosters the reincorporation of the industrial wastes into production processes to reduce disposal costs and environmental impact, while providing eco-friendly sources of high-value-added products [84,85].

Moreover, Salazar et al. studied avocado seed, peel, and dry pulp, reporting, respectively, the presence (%wt) of carbohydrates (43–85%, 44–84%, and 32%), lipids (2–4%, 2–6%, and 55%), proteins (3–9%, 3–8%, and 7.5%), and minerals (2–4%, 2–6%, and 6%). This biochemical composition allows for the design of biobased polymers, which have emerged with an advantage over conventional materials due to their biodegradability and renewability [83]. Carbohydrates can be used in the energy and fuel industry (e.g., bioethanol). Although seeds and peels are being prioritized, pulp residues are also sources of monounsaturated fatty acids, oleic acid, palmitic acid, tocopherols, tocotrienols, phytosterols, carotenoids, and polyphenols, which may potentially exhibit pharmacological activity in human beings [86].

Páramos et al. developed a method for oil extraction and the recovery of bioactive compounds from Brazilian and Mexican avocados, using only their waste materials (peels and seeds). The research group used a Soxhlet system with hexane, ethanol, and ethyl acetate as solvents. In addition, they employed as cosolvents supercritical CO_2_ (SC–CO_2_), ethanol, and ethyl acetate. The results demonstrated the greatest extraction efficiency for the system that used ethanol as a solvent and supercritical carbon dioxide as a cosolvent, where oil extraction yielded up to 10.3 wt% for the Brazilian seeds and 14.0 wt% for the Mexican peels [16].

Another method for optimizing the recovery of bioactive compounds was developed by Figueroa et al. In this research, phenolic compounds from avocado peels were extracted using pressurized liquid extraction with safe non-toxic solvents. The research group defined the conditions of 200 °C as the extraction temperature and a solvent system composed of 1:1 ethanol/water. More than 47 phenolic compounds were isolated in the obtained extracts [87].

In another approach, Ortiz et al. evaluated the effect of different extraction methods (microwave extraction, hexane extraction, microwave–hexane combined extraction, and acetone extraction) on the recovery of fatty acids, as well as on the physicochemical properties of avocado oil. The study suggests that the highest fatty acid yield is obtained when using the combined microwave–hexane extraction. In addition, oil physicochemical properties are affected mainly by solvents rather than by microwaves [88].

The biorefinery approach for avocado by-products can also have applications in the food industry. Permal et al. used dried avocado wastewater generated by cold-pressed oil production to assess its suitability to be sprayed as a natural food preservative in cooked pork sausages. The wastewater contained 6.3 wt% of lipids, and its antioxidant activity increased after spray drying, being as effective as synthetic antioxidants to inhibit lipid oxidation [89].

## 3. High-Value Products Obtained from Avocado Wastes

### 3.1. Biofuels and Bioenergy

The importance of fossil fuels is well known worldwide, as they are the most common source of energy commercialized. However, their production limitations and the depletion of these fossil sources cause their price to increase on a regular basis [90]. Considering the significant amount of waste obtained by the avocado industry, the production of bioenergy and biofuels from these are regarded as possible alternatives to fossil fuels in the short-term [91].

**Figure 3:**
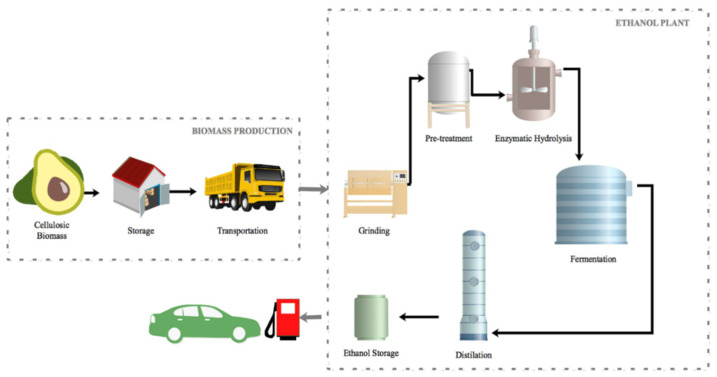
Bioethanol production from avocado’s cellulosic biomass. Reprinted with permission from Karagoz P et al. Lignocellulosic ethanol production: Evaluation of new approaches, cell immobilization and reactor configurations. *Renewable Energy*. Copyright (2019) Elsevier [93].

In the biorefinery approach, biomass conversion into biofuels and biochemicals represents a promising and valuable way to mitigate global warming and diversifying energy sources [92]. In that sense, cellulosic biomass from the avocado seed and peel can be used for bioethanol production through fermentation (Figure 3) [93,94]. Woldu et al. employed powdered avocado seed hydrolysate wastes for producing bioethanol. The optimum fermentation conditions were achieved using *Saccharomyces cerevisiae* for 3 days, and a pH medium of 5.5 at 30 °C [95]. Additionally, Pratywi et al. reported the use of avocado seed powder to obtain starch that can be degraded by simple heating. This degradation can be considered a suitable method to recover glucose for bioethanol production [96].

This approach can also be employed for obtaining other substances with industrial importance, such as butanediol. However, there is still a major challenge regarding the optimization of the lignocellulosic raw material conversion to ethanol [97].

The latest efforts and research are focused on solving this lack of technology. In this sense, Gu et al. proposed the use of SC–CO_2_ explosion pretreatment as a green solvent to treat the biomass before enzyme hydrolysis [98]. This solvent is of great interest due to its natural character, non-toxicity, and non-flammability within the operating conditions [99]. In addition, conventional enzyme processes require large amounts of water and energy, while SC–CO_2_ is designed to reduce and optimize the energy used [98].

To date, some studies have declared it possible to obtain biodiesel from avocado seed through transesterification, which simplifies the process due to the low content of free fatty acids and the presence of triglycerides [81,91,100]. Colombo et al. determined that the fruit wastes have the potential to be transformed into biofuel. Pyrolysis and torrefaction allow obtaining liquid fuel, while for solid fuel, further thermal treatment is required to reduce part of the volatile liquid [17].

### 3.2. Drugs and Bioactive Compounds

It is well known that avocado contains many bioactive compounds that make this fruit a potential source for different drug candidates, which has attracted the attention of the pharmaceutical industry [101]. The fruit is remarkable because of its antioxidant activity, especially aqueous and alcoholic peel extracts [81]. A study carried out by Alkhalaf et al. proved significant antioxidant activity present in the seed as well [102].

Although seeds and peels are being prioritized, pulp residues are also sources of relevant therapeutic compounds. In these wastes, different studies have reported the presence of monounsaturated fatty acids, metabolites (e.g., oleic, palmitic acid), and tocopherols, tocotrienols, phytosterols, carotenoids, and polyphenols, which may potentially exhibit pharmacological activity in human beings [18,86].

Moreover, gastric, anti-inflammatory, and antimicrobial activities obtained from avocado wastes are currently being studied [103,104]. Athaydes et al. exposed the possible use of this product to prevent gastric ulcer disease in mice [105]. In this study, its efficacy relies on lowering mediators’ production, such as IL-6 and PEG_2_, known as proinflammatory factors [105,106]. In another approach, Dabas et al. confirmed this anti-inflammatory activity by anin vitroassay. The research group used an extract of the avocado seed on lipopolysaccharide (LPS)-induced inflammatory responses of murine macrophages, which revealed a reduction in the production of the inflammatory factors significantly [107].

The antibacterial activity is regarded with great interest by the scientific community. According to current studies, antibiotic-resistant bacteria will be the leading cause of death in the year 2050 and are becoming a real threat to human health [108]. Thus, it is imperative to develop new antibiotic drugs using natural resources, such as avocado wastes, which have exhibited antibacterial and antifungal activity.

Amado et al. prepared peel, seed, and pulp ethanolic extracts to evaluate the antimicrobial activity on *Staphylococcus aureus*, *Bacillus cereus, Escherichia coli*, and *Salmonella typhi*. The results determined that the peel showed a better bactericidal and bacteriostatic effect over the Gram-positive. However, the different ethanolic extracts also demonstrated activity against Gram-negative bacteria [109].

Villarreal et al. and Salinas et al. evaluated the inhibitory activity of avocado acetogenins (i.e., fatty acid derivatives) used against *Listeria* *monocytogenes*. In these studies, the minimum inhibitory concentration (MIC) showed a bactericidal effect probably due to increased membrane permeability and lytic effects. Therefore, the test with *L. monocytogenes* was successful, decreasing initial growth of bacteria. In addition, the results showed a significant inhibition activity against Gram-positive and Gram-negative strains [110,111].

Furthermore, caffeoylquinic acid, a high-interest product, is present in the fruit as well. This substance possesses therapeutic properties due to its pharmacological activity as an anti-inflammatory, antiviral, anticancer, and antidiabetic molecule [112,113]. These properties, combined with the possibility of obtaining biopolymer from avocado wastes, represent a great opportunity for developing advanced drug delivery systems that can improve the bioavailability of these compounds and, thus, their therapeutic effect [17].

### 3.3. Biopolymers

Biotechnological innovations have developed high-value-added processes and biomaterials with a significant impact on regenerative medicine and for the design of biomedical devices. In order to successfully achieve the former, a convergence between bioactivity, biocompatibility, biodegradability, and good mechanical properties is necessary for the generation of new biomaterials [114,115].

Among the materials used for the design of medical devices, plastics such as polyethylene (PE) and polypropylene (PP) are widely used. However, the medical device industry demands the designing and engineering of new materials made from biomass as raw material. The biobased polymer approach takes advantage of polysaccharides in the avocado peel and seeds, where chemical and/or microbiological treatments can transform these wastes into biomaterials such as polylactic acid (PLA), nanocellulose fibers (NCFs), and polyhydroxybutyrates (PHBs) [7].

#### 3.3.1. PolylacticAcid

Avocado wastes can be pretreated with filamentous fungi or Gram-positive bacteria such as *Lactobacillus* *delbruekii.* The solid or liquid bioprocesses involved can generate lactic acid, which allows for obtaining PLA through a process that seeks to reduce the impact on the environment, improves processibility, and results in energy savings [116]. Coban et al., in the case of *Rhizopus oryzae*, described how fermentation with fungi may be most effective due to high concentrations of starch in the remaining biomass. Once the lactic acid has been produced and extracted, PLA polymerization is carried out [117].

#### 3.3.2. Polyhydroxybutyrate (PHB)

PHB is a biodegradable and biocompatible thermoplastic. It belongs to the polyhydroxyalkanoate family (PHAs), demonstrating an enormous potential to replace petroleum-based plastics [118,119]. According to Getachew et al., a wide variety of bacteria such as *Bacillus* spp. can use biomass waste to perform its synthesis and production as an intracellular storage material, also known as inclusion bodies. Thus, it is possible to obtain this plastic from avocado waste material [120].

This biopolymer can be positively used for cell growth, tissue scaffolding for nerve and bone regeneration, controlled-release drug delivery, surgical sutures, cartilage, and cardiovascular support, thermo gels, and wound dressing [118,121]. Moreover, PHB is used in nanocomposites associated with other plastics (e.g., PLA), which improves the mechanical properties and makes them stronger [122]. Regarding this, Kai et al. described an improved tensile strength and elongation when these plastics are combined [123].

#### 3.3.3. Starch Applicability

Starch is a renewable biopolymer material that is entirely biodegradable, easy to handle, and widely available in nature, such as in the avocado [124,125]. Seeds have shown to contain a large amount of starch that can reach over 90%. The recollection and processing of waste avocado seeds can be a new source of starch and provide income opportunities, since this macromolecule is widely employed in different fields for textiles, pharmaceutical excipients as a binder and adhesive, food processing, beverages, and more. In addition, many biopolymers based on starch can be obtained [126,127].

Hendra et al. used avocado starch to produce a plastic film since this material is composed of approximately 73% of amylopectin, making it a suitable raw material for the application. The manufacturing of the plastic film followed the standard method consisting of heating the mixture of starch filler solution until gelatinization [128]. Although starch bioplastics allow for obtaining a hydrophilic material, the mechanical properties tend to be weak. Therefore, it is necessary to continue researching these materials to improve their physicochemical and mechanical properties [129,130].

Araújo et al. evaluated avocado by-products to recover starch through microwave-assisted extraction (MAE). The obtained biopolymer exhibited high solubility, low water absorption capacity, a non-granular structure with particles smaller than 2 µm, and polydispersity at different sizes. Therefore, starch extraction resulted in being suitable and feasible by MAE technology when using avocado seed [131].

#### 3.3.4. Nanocellulose Fibers (NCFs)

Likewise, a high-value product obtained from avocado seed and peel is NCFs. As described before, cellulose is one of the most abundant polymers in biomass waste [132]. Thus, avocado wastes represent a great source of cellulose for the production of these nanofibers with outstanding properties due to their high surface area, rheological behavior, water absorption, and high bending strength (~10 GPa). In addition, NCFs are used in the food and medical industry since these are not cytotoxic nor genotoxic. [133,134].

Regarding the food industry, NCFs are used as stabilizing agents (i.e., a functional ingredient in foods and food packaging) [133]. On the other hand, in the medical industry, they can be used as scaffolds for cellular culture, drug excipients and drug delivery, and enzyme immobilization. Additionally, they can be used in the design of macroscopic materials such as catheters, skin and bone repair, and antimicrobial materials [134].

## 4. Nanotechnology

Nanotechnology is increasingly gaining popularity because it plays an essential role in many fields, such as pharmaceuticals, medicine, material engineering, and environmental remediation [135]. This technology offers a wide range of opportunities to develop new products and processes for designing high-value-added products.

There are multiple applications in the agro-industrial area, such as in pest control by designing nanomaterials. Avocado seed and peel can be used for extracting nanocellulose, which can work as a new packing material and as a carrier of antimicrobial agents extracted from the same fruit waste [136,137]. Additionally, phenols and essential oils are added to nanocomposites to improve food materials’ lifespan and are used for the production of nanoemulsifiers, which have advantages over conventional emulsifiers [138].

Another incredible research area is the green synthesis of nanoparticles with multiple applications according to their properties. For example, the biosynthesis of copper, silver, and gold nanoparticles using fruit by-products represents great potential for biomedical applications due to the nanoparticles’antimicrobial and antioxidant properties [135,139].

## 5. Challenges and Future Perspectives

Advanced research is looking for the development of renewable lignin-based biopolymers, for which novel technologies are required by engineering plant feedstocks to achieve structural homogeneity in the separation and purification procedures. Although this irreversible trend represents an eco-friendly process, the growing of agricultural products without proper management of soils and pesticides use may cause the depletion of soil nutrients. In addition, biopolymers’ conversion into useful materials requires chemical and biological additives, so improvements at all stages of their life cycle should be done to preserve environmental integrity [74,85,140].

Moreover, nanotechnology will play an important role in taking advantage of all these biopolymers. Developing novel platforms based on hybrid biopolymeric systems with applications in nanomedicine, environmental preservation, and food packaging will be necessary for years to come [141]. More joint efforts from multidisciplinary and multi-industry research, investment, and social commitment are needed to make all the proposed technologies and bioprocesses viable and for agro-industries to migrate towards these biorefinery models.

## 6. Concluding Remarks

An integrated biorefinery approach applied to avocado excessive wastes allows for reintroducing these into the production chain. The transformation of waste biomass into high-value-added products provides society with the opportunity to develop renewable fuels, biobased materials, and chemicals for different pharmaceutical, biotechnological, nanotechnological, agro-industrial, and engineering applications. At the same time, LCB plays an irreplaceable role in human society’s attempt at sustainable development with a minimum impact on the environment. The valorization of avocado wastes, as well as any other natural by-product, holds potential for mid-term replacement of fossil carbon fuels in a biobased economy. This could provide a suitable strategy to manage natural resource depletion and to create sustainable economic growth from a bioeconomic perspective.
